# Case Report: Pulmonary Leptospirosis Misdiagnosed as COVID-19

**DOI:** 10.4269/ajtmh.21-1102

**Published:** 2022-05-16

**Authors:** Jean-Marie Turmel, Claude Olive, Bastien Bigeard, Sylvie Abel, Rishika Banydeen, Laura Daoud, Pierre-Marie Fayolle, André Cabié, for the COVID-19 Study Group of the University Hospital of Martinique

**Affiliations:** ^1^ Department of Tropical and Infectious Diseases, Centre Hospitalier Universitaire de Martinique, Fort de France, France;; ^2^ Bacteriology Laboratory, Centre Hospitalier Universitaire de Martinique, Fort de France, France;; ^3^ Department of Clinical Research, Centre Hospitalier Universitaire de Martinique, Fort de France, France;; ^4^ Intensive Care Unit, Centre Hospitalier Universitaire de Martinique, Fort de France, France;; ^5^ Centre Hospitalier Universitaire de Martinique, Fort de France, France

## Abstract

We report the case of an 83-year-old woman with acute, febrile respiratory failure resulting from interstitial pneumonia that required high-flow oxygen therapy. This clinical picture, associated with the ongoing epidemiological situation, initially guided us toward a diagnosis of COVID-19. Based on SARS-CoV-2 reverse transcription–polymerase chain reaction negativity and the absence of anti-SARS-CoV-2 antibodies, a search for a differential diagnosis was conducted that led us to conclude a diagnosis of severe pulmonary leptospirosis This case highlights the need to engage in early discussions about differential diagnoses, including neglected tropical and subtropical diseases, during the COVID-19 era.

## INTRODUCTION

SARS-CoV-2 infection has generated a worldwide pandemic since early 2020. In a pandemic situation, the proportion of COVID-19 compared with other infectious diseases increases among primary care patients. This epidemiological trend modification can lead to diagnostic errors, in particular when clinical, biological, or radiological similarities exist. We report a case of severe pulmonary leptospirosis misdiagnosed as severe COVID-19.

## CASE REPORT

In the French Caribbean island of Martinique, the second COVID-19 pandemic wave occurred between September 1 and December 31, 2020. During this period, 290 patients were treated in our center, a tertiary university hospital.

On December 6, 2020, an 83-year-old woman without significant medical history presented to the emergency room with dyspnea. The patient’s symptoms appeared on December 2 (day 1) and included myalgia, arthralgia, and diarrhea. Three days later, she was dyspneic and had a fever, which led her to be admitted to our hospital.

On admission, the patient’s body temperature was 38.8°C, with an oxygen saturation at ambient air of 88% and polypnea at 40 cycles/min. The patient was dyspneic when talking or carrying out low-level physical activity. Clinical examination revealed bilateral crackles at the pulmonary bases, with the absence of jaundice, rash, and conjunctival suffusion. Her mental status was deemed normal.

Laboratory exams showed lymphopenia at 140 lymphocytes/mm^3^, thrombocytopenia at 101,000 thrombocytes/mm^3^, and a serum creatinine level of 91 µmol/L (normal value, 55 µmol/L). The patient’s blood urea nitrogen level was 11.6 mmol/L, and aspartate aminotransferase and alanine aminotransferase were at 160 and 62 U/L respectively. Her bilirubin level was normal, C-reactive protein was at 350 mg/L, and B-type natriuretic peptide was normal at 105 pg/mL (Table 1).

**Table 1:** Patient’s blood test and microbiological results during hospitalization

Results	Day 5* (12/6/2020)	Day 7* (12/8/2020)	Day 8* (12/9/2020)	Day 10* (12/11/2020)
Leukocytes (cells/mm^3^)	1,650	10,120	11,190	12,540
Neutrophils/mm^3^	1,490	9,150	–	–
Lymphocytes/mm^3^	140	680	–	–
Platelets/mm^3^	101,000	81,000	109,000	138,000
C-reactive protein (mg/L)	350.2	395.1	–	153.7
Creatinine (µmol/L)	91	60	–	56
Blood urea nitrogen (mmol/L)	11.6	7.9	–	13.3
Kalemia (mmol/L)	2.6	3.4	–	3.9
Aspartate aminotransferase (U/L)	160	61	35	–
Alanine aminotransferase (U/L)	62	56	41	–
Total Bilirubin (mmol/L)	15.9	8	12.7	13.4
NP SARS CoV-2 RT-PCR	Negative	Negative	–	–
Serum SARS CoV-2 IgG and IgM (ELISA)	Negative	–	–	Negative
Serum *Leptospira* RT-PCR	Positive	–	Negative	Negative
Serum *Leptospira* IgM (ELISA)	Negative	–	Positive	–

NP = nasopharyngeal; RT-PCR, reverse transcription–polymerase chain reaction.

* After symptom onset. Symptom onset (day 1) was December 2, 2020.

**Figure 1.**
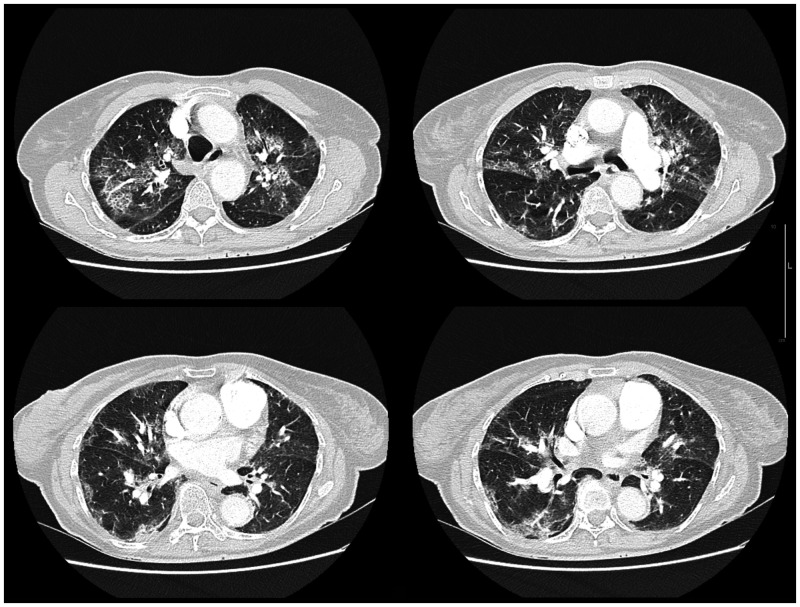
Computed tomographic chest scan at admission, 5 days after symptom onset.

A first SARS-CoV-2 reverse transcription–polymerase chain reaction (RT-PCR) test was performed on a nasopharyngeal sample on day 5 of symptoms and was negative. Influenza, respiratory syncytial virus, metapneumovirus, and rhinovirus PCR tests were also negative, as were serum dengue virus RT-PCR and dengue serology. *Legionella pneumophila* (serogroup I) urinary antigen was equally absent. Computed tomography (CT) of the chest was performed on admission and revealed ground-glass opacities, with a distribution that was bilateral and mixed: peripheral and peri-bronchovascular. About 25% of the lung parenchyma was affected (Figure 1).

At this stage, the suggested diagnosis was severe COVID-19 pneumonia, with a false-negative RT-PCR. Rapidly, on day 6, the patient’s respiratory condition worsened, requiring admission to the intensive care unit (ICU) to increase oxygen therapy. The administered ICU treatment combined high-flow oxygen therapy with OPTIFLOW™ (Fisher & Paykel Healthcare, Auckland, New Zealand) and corticosteroid therapy with dexamethasone (6 mg/d). No antibiotic therapy was introduced, based on the negativity of the initial microbiological and CT examinations. Respiratory parameters improved on day 8, and high-flow oxygen therapy was weaned. The patient left the ICU on day 9 and was transferred to the infectious disease ward.

Faced with the lack of microbiological documentation, investigations were extended. SARS-CoV-2 RT-PCR tests on a nasopharyngeal sample were repeated on days 5 and 7 after symptom onset and were again negative. Anti-SARS-CoV-2 IgM and IgG antibody serologies performed on days 5 and 10 were equally negative.

The medical team’s hunch was then to test for the presence of leptospirosis, a tropical disease endemic to the island. Leptospirosis is known to have a strong seasonality, with incident cases expected mostly during the rainy season (the last 3 months of the year for Martinique). Leptospirosis serology performed on day 8 with ELISA (OnSite™ *Leptospira* IgG/IgM Combo Rapid Test) showed the presence of anti-*Leptospira* IgMs. IgM presence was confirmed with another ELISA test (Hurstbridge), with a titer of 1:6,400. On the same blood sample, a microagglutination test (Martin et Pettit Microagglutination test) did not, however, allow the identification of a specific *Leptospira* serovar, probably because of the precocious timeliness of sample collection. A *Leptospira* RT-PCR test was also performed on the same day (day 8) and was negative. This RT-PCR was carried out on an Applied Biosystems™ 7500 Real-Time PCR Systems (Thermo-Fisher Scientific, Waltham, MA) using the DNA-binding dye technique (SYBR Green), with the primer set consisting of LFB1-F (5′-CATTCATGTTTCGAATCATTTCAAA-3′) and LFB1-R (5′-GGCCCAAGTTCCTTCTAAAAG-3′). The entire process is detailed further by Merien et al.^1^ Faced with a positive *Leptospira* serology on day 8 and a concordant clinical picture, a *Leptospira* RT-PCR test was performed retrospectively on a blood sample collected on day 5, and it turned out to be positive. On the basis of these microbiological confirmations and the patient’s previous CT results, the diagnosis of leptospirosis-induced pneumonia was retained: pulmonary leptospirosis. These findings triggered the start of antibiotic therapy with amoxicillin on day 10, despite clinical improvement. Steroid therapy was continued. From then on, the patient’s clinical condition improved progressively. Renal function recovered without needing hemodialysis; diuresis was always preserved. A retrospective investigation yielded that the patient might have been exposed to *Leptospira *via rats present in her garden.

The patient was discharged from the hospital to her home on December 15, 2020, 12 days after admission. Before discharge, the patient provided informed oral consent for the use of her medical data for research purposes.

## DISCUSSION

Pulmonary leptospirosis and COVID-19 pneumonia share many similarities. Those similarities in clinical presentation, combined with the ongoing COVID-19 pandemic wave at the time, led to the patient’s misdiagnosis. Indeed, flu-like symptoms, acute respiratory distress, a high C-reactive protein level, and CT-identified peripheral ground-glass opacities are signs that are strongly suggestive of either one of these two diseases. As such, although leptospirosis is endemic to tropical Martinique, its diagnosis was finally evoked, only when faced with the negativity of first-intention microbiological assessments for respiratory disease. Pulmonary leptospirosis diagnosis was confirmed through the combination of two test results: a positive PCR on the day 5 blood sample, and IgM-positive ELISA serology on day8. This thus rules out the hypothesis of a random false-positive test result. As for the negative PCR result for *Leptospira* on the day 8 blood sample, it can be explained by the known fact that leptospiremia occurs during the first acute disease stage before symptom onset, and usually lasts 7 days at most.^2^ Leptospirosis severity was established based on the presence of the following criteria: severe respiratory failure, initial renal injury with hypokalemia, and elevation of transaminases.

Leptospirosis is a globally distributed, neglected zoonotic infection affecting mainly subtropical and tropical areas, and more rarely temperate areas. It is caused by spirochete bacteria of the genus *Leptospira*. Clinical presentation can be varied, ranging from a subclinical disease to multiple-organ failure. According to O’Neil et al.,^3^ pulmonary complications occur in 20% to 70% of cases. They are characterized by intra-alveolar hemorrhage and lesional pulmonary edema. The pathogenesis features leading to alveolar hemorrhage are not yet fully understood. *Leptospira* adhesion could be possible thanks to its attachment capacity to cadherins. One of the induced damage mechanisms could be toxin-mediated destruction; endotoxin activity of the outer membrane of spirochetes and production of exotoxin could lead to septal capillary injury.^4^^,^^5^ Another presumed mechanism is a host immune response, with elevated levels of inflammatory cytokines, as reported in patients with leptospirosis.^2^^,^^6^^,^^7^

Leptospirosis-linked respiratory symptoms may manifest either as mild symptoms (dyspnea, cough, hemoptysis) or immediately as acute respiratory failure. These symptoms most commonly appear in a context of fever or flu-like illness, between days 4 and 6 after disease onset.^4^^,^^6^ In a pulmonary leptospirosis case series by Gouveia et al.,^8^ hemoptysis was an uncommon symptom at admission (15%), whereas pulmonary hemorrhage was visible during intubation for only 40% of patients. Non-respiratory manifestations were similar between those with and without pulmonary leptospirosis. Chest radiography can be normal despite lung damage.^9^ CT is more sensitive for the detection of intra-alveolar hemorrhage signs. With high-resolution CT, bilateral ground-glass opacities are the most frequent abnormalities. Areas of consolidation or crazed paving patterns have also been described.^4^

*Leptospira* remains susceptible to many antibiotics, including beta-lactams. Penicillin or doxycycline are the first-line antibiotic therapies usually used. The benefit of antibiotic therapy is, however, controversial regarding mortality and symptom duration.^10^ It might only have a benefit during the early days of the disease, mainly in complicated forms. Complications of leptospirosis being partly immune mediated, anti-inflammatory agents could contribute to the treatment. Adjunctive steroid treatment has also been discussed by some authors, with the sole open-label randomized clinical trial published until now, concluding on its ineffectiveness.^11^^,^^12^

The case fatality rate of pulmonary leptospirosis remains very high (> 50%). Pulmonary involvement is thus a pejorative prognostic factor of leptospirosis.^4^^,^^6^^,^^8^

## CONCLUSION

This case report raises awareness about the risk of misdiagnosis during COVID-19 pandemic phases, particularly relating to pulmonary leptospirosis. The latter’s prognosis is serious, and early antibiotic administration is recommended. In the presence of an evocative clinical profile, combined with negative SARS-CoV-2 RT-PCR test, it should be sought systematically in endemic areas for leptospirosis or in travelers returning from these areas.
